# On the Efficacy of ZnO Nanostructures against SARS-CoV-2

**DOI:** 10.3390/ijms23063040

**Published:** 2022-03-11

**Authors:** Maria Chiara Sportelli, Margherita Izzi, Daniela Loconsole, Anna Sallustio, Rosaria Anna Picca, Roberto Felici, Maria Chironna, Nicola Cioffi

**Affiliations:** 1 Chemistry Department, University of Bari Aldo Moro, Via Orabona, 4, 70126 Bari, Italy; maria.sportelli@uniba.it (M.C.S.); margherita.izzi@uniba.it (M.I.); rosaria.picca@uniba.it (R.A.P.); 2 Department of Biomedical Sciences and Human Oncology-Hygiene Section, University of Bari Aldo Moro, Piazza Giulio Cesare, 11, 70124 Bari, Italy; daniela.loconsole@uniba.it; 3 Azienda Ospedaliero-Universitaria Consorziale Policlinico di Bari, Piazza G. Cesare, 11, 70124 Bari, Italy; annasallustio@libero.it; 4 CNR-SPIN, Area Della Ricerca di Tor Vergata, Via del Fosso del Cavaliere, 100, 00133 Rome, Italy; roberto.felici@spin.cnr.it

**Keywords:** zinc oxide, nano-antimicrobials, green synthesis, antiviral, COVID-19, nucleocapsid protein, nasopharyngeal swab, SARS-CoV-2

## Abstract

In 2019, the new coronavirus disease (COVID-19), related to the severe acute respiratory syndrome coronavirus (SARS-CoV-2), started spreading around the word, giving rise to the world pandemic we are still facing. Since then, many strategies for the prevention and control of COVID-19 have been studied and implemented. In addition to pharmacological treatments and vaccines, it is mandatory to ensure the cleaning and disinfection of the skin and inanimate surfaces, especially in those contexts where the contagion could spread quickly, such as hospitals and clinical laboratories, schools, transport, and public places in general. Here, we report the efficacy of ZnO nanoparticles (ZnONPs) against SARS-CoV-2. NPs were produced using an ecofriendly method and fully characterized; their antiviral activity was tested in vitro against SARS-CoV-2, showing a decrease in viral load between 70% and 90%, as a function of the material’s composition. Application of these nano-antimicrobials as coatings for commonly touched surfaces is envisaged.

## 1. Introduction

Zinc is known to exert antiviral and antibacterial activity, as well as regulate the inflammatory response [1]. Preliminary experimental evidence suggests that zinc ions may be beneficial for the treatment of COVID-19 [2,3], and ZnONPs have specifically demonstrated good antiviral activity against other viruses, including influenza A(H1N1) [4] and herpes simplex type 1 [5]. The mechanism of action of ZnONPs against coronaviruses is not known; some hypotheses propose the ability of Zn^2+^ ions to bind proteins and other biological macromolecules, modulating their activity. Among them, two essential proteins for the CoV lifecycle are the RNA-dependent RNA-polymerase (RdRp), which is necessary for proper viral replication, and the 3C-like proteinase (3CL), which is necessary for polyprotein processing and essential for viral replication [6]. Some preliminary studies have demonstrated the potential inhibition of angiotensin-converting enzyme 2 (ACE2) function by traces of zinc ions in an intercellular environment, which could restrict the ability of SARS-CoV-2 to infect cells [7]. In fact, SARS-CoV-2 spike proteins have a 20-fold higher binding affinity to human ACE2 receptors than other CoVs, resulting in greater infectivity [8]. Zinc ions can block the ability of ACE2 to metabolize substrates via a dose-dependent response, at extremely low concentrations (10 µM) [9], suggesting that Zn^2+^ could prevent the interaction between SARS-CoV-2 spike protein and ACE2 [7]. This zinc concentration does not exceed its physiological one; human plasma zinc ranges from approximately 10 to 18 μM [10]. On the basis of this evidence, we can infer that metal oxide NPs (ZnO, iron oxides, TiO_2_, etc.) mainly act through the release of cations in solution [11]. This is quite different from what is hypothesized to be the prevalent bioactivity mechanism for other nanosized metal NPs, which mainly exert their antimicrobial activity via the production of reactive oxygen species (ROS) [12]. The above-presented hypotheses pave the way for the potential use of ZnONPs both as a coating for self-sanitizing surfaces and as an antiviral agent, interfering with the viral replication cycle.

In this paper, we report the in vitro antiviral activity of ZnONPs against SARS-CoV-2 over a 24 h timespan. ZnONPs were synthesized using a green approach. The nanomaterial was characterized, from both the morphological and the spectroscopic point of view. The possibility of embedding ZnONPs in polymeric films, for their final application as an antiviral coating, was also foreseen.

## 2. Materials and Methods

### 2.1. Materials

Zinc and platinum sheets (1 mm thick, 99.99+%) were purchased from Goodfellow LTD and cut into 2 × 1 cm^2^ pieces. Sodium hydrogen carbonate (NaHCO_3_, purum p.a., 99.0%), 2-propanol (IPA, Chromasolv^®^ Plus, for HPLC, 99.9%), hydrochloric acid (HCl, ACS reagent, 37%), cetyltrimethylammonium bromide, (CTAB, 95%), poly-diallyl-(dimethylammonium) chloride (PDDA, average MW 200,000–350,000, 20 wt.% in H_2_O), poly (sodium 4-styrenesulfonate) (PSS, average Mw ∼70,000, powder), polyethylene oxide (PEO, average M_w_ 100,000), ZnSO_4_·7H_2_O salt, boric acid (≥99.9%), sodium hydroxide, 2-(*N*-morpholino)ethanesulfonic acid sodium salt (MES, ≥99.5%,), tris(hydroxymethyl)aminomethane hydrochloride (TRIS), and ethanolamine (EA, ≥99.0%) were purchased from Merck Sigma–Aldrich (Milan, Italy). Zincon sodium salt (2-[5-(2-hydroxy-5-sulfophenyl)-3-phenyl-1-formazyl]benzoic acid monosodium salt) was purchased from Alfa Aesar (Kandel, Germany). Aluminum oxide (Al_2_O_3_, purum p.a., 99.7%), for the mechanical polishing of zinc sheets, was obtained from Fluka Chemicals (Italy). Milli-Q water was used in all experiments, including the preparation of all buffers and standard solutions. Nasopharyngeal swabs collected in COPAN^™^ transport medium (COPAN ITALIA spa, Brescia, Italy) from patients with a suspected SARS-CoV-2 infection were used for laboratory tests. Ethical approval was not required because the samples were analyzed as part of routine diagnostic tests. All procedures were carried out in accordance with the Declaration of Helsinki, as revised in 2013, for research involving human subjects. The data were deidentified; therefore, the need for informed consent was waived. Samples were processed at the Laboratory of Molecular Epidemiology and Public Health of the Hygiene Unit of the Policlinic Hospital of Bari, which is the regional reference laboratory for surveillance and diagnosis of SARS-CoV-2 infections. Viral serial dilutions were prepared in Dulbecco’s phosphate-buffered saline w/o calcium and w/o magnesium (DPBS) (EuroClone, Milan, Italy).

### 2.2. Synthesis and Characterization of ZnONPs

ZnONPs were here synthesized using a green and scalable electrochemical procedure developed in our laboratories [13]. Aqueous electrosynthesis was conducted under mild galvanostatic conditions in the presence of both cationic and anionic stabilizers (CTAB, PSS, PDDA). A three-electrode cell was utilized, lying on a Zn sheet as the working electrode, a Pt sheet as the counter electrode, and Ag/AgCl (KCl sat.) as the reference electrode. Zn plates were initially polished using sandpaper, and then using alumina slurries at decreasing granulometry. After sonication, alternating MilliQ water and IPA, they were immersed in 1 M HCl for 30 s to ensure activation. A current density j = 10 mA/cm^2^ was applied, with a CH-1140b potentiostat–galvanostat (CH Instruments, Bee Cave, TX, USA). The process was performed at room temperature, except for the CTAB stabilizer, where the synthesis was conducted at 80 °C in a mineral oil bath [14]. The electrolytic medium was composed of 0.1 M CTAB or 1 g/L PSS or PDDA, dissolved in 30 mM NaHCO_3_ aqueous solution. After the synthesis, the colloidal dispersion was centrifuged at 6000 rpm for 30 min. The precipitate was dried overnight at 70 °C (120 °C for PDDA). The obtained powder underwent calcination at various temperatures in a muffle furnace for 1 h; specifically, we used 300 °C for CTAB-stabilized ZnONPs and 600 °C for PSS ones. No calcination was performed for PDDA-ZnONPs.

The ZnONP morphology was studied by transmission electron microscopy (TEM, FEI Tecnai 12, Hillsboro, OR, USA; high tension: 120 kV; filament: LaB_6_). Selected area electron diffraction (SAED) measurements were performed using the same conditions, in dark field. Nanoparticle size analysis and SAED pattern studies were performed with ImageJ software (http://imagej.nih.gov/ij/, accessed on 10 February 2022).

X-ray powder diffraction (XRD) spectra were collected using a D/max-Rigaku diffractometer recently fully refurbished by DFP technologies, Trento, Italy. The spectra were collected using as X-ray source a Cu sealed tube, operating at 25 kV and 25 mA of emission current, in a 𝜗–2𝜗 geometry. The recorded 2𝜗 interval was in the range 30°–80° with a step of 0.05°. The main diffraction peaks of ZnO appeared in this interval. The counting time was 1 s for each point, and five spectra were evaluated in order to obtain satisfactory results.

NP spectroscopic characterization was performed by a Shimadzu UV-1601 double-beam spectrometer with 1 cm quartz Suprasil^®^ cuvettes.

Bulk chemical composition of the powders was obtained by attenuated total reflectance infrared (ATR-FTIR) analysis on a Spectrum Two FTIR spectrometer (PerkinElmer, Milan, Italy) with a resolution of 2 cm^−1^ scanning from 4000 to 400 cm^−1^. A total of 32 scans were averaged; background spectra were acquired against air, and spectral baseline subtraction was performed using the instrument software.

The ζ-potential and dynamic light scattering (DLS) measurements were carried out using a Zetasizer-Nano ZS from Malvern Instruments (Rome, Italy). Measurement details were reported elsewhere [15]. DLS characterization was performed on the different kinds of ZnONPs dispersed in DPBS.

### 2.3. SARS-CoV-2 Nucleocapsid Protein Antigen Quantification

Nasopharyngeal swabs were tested using the chemiluminescence enzyme immunoassay (CLEIA) antigenic test (LumiPulse SARS-CoV-2 Ag kit, Fujirebio, Inc., Tokyo, Japan). To perform the test, samples were centrifuged at 2000× *g* for 10 min, and the supernatant was used for analysis with the Lumipulse SARS-CoV-2 Ag kit (Fujirebio), which was then read by the Lumipulse G1200 automated immunoassay analyzer (Fujirebio). For positive samples, the initial concentration of SARS-CoV-2 nucleocapsid protein antigen was quantitatively measured, and sample serial dilutions were prepared in DPBS. Each dilution was exposed to ZnONPs at a concentration of 10 g/L or 1 g/L. After 24 h, biological samples were filtered by nylon 0.22 µm syringe filters and analyzed again through antigenic test.

### 2.4. Preparation of ZnO-Based Coatings for Touching Surfaces

CTAB-, PSS-, and PDDA-capped ZnONPs suspended in water were mixed to a 5 g/L PEO solution, at a concentration of 10 g/L. PEO was dissolved in isopropyl alcohol, under stirring at 60 °C for 2 h. Then, the dispersion was added to the polymeric solution, and the mixture was stirred for a further 30 min. The resulting composite was deposited by spin-coating, with a rotation program of 500 rpm for 30 s and 5000 rpm for 60 s. A volume of 500 μL was deposited on 20 × 20 mm^2^ substrates.

### 2.5. Surface Chemical Composition of ZnO-Based Coatings by X-ray Photoelectron Spectroscopy

X-ray photoelectron spectroscopy (XPS) characterization was carried out on PEO coatings embedding CTAB-, PSS-, and PDDA-ZnONPs, prepared as reported in Section 2.4, to evaluate their surface chemical composition. A Versaprobe II spectrometer (PHI, Chanhassen, MN, USA) with a monochromatic Al K_α_ source (spot size 200 µm) was used. Identification of main elements was performed acquiring survey scans, and their quantification in terms of atomic percentage was carried out on high-resolution spectra for each element using Multipak processing software (v. 9.9.0.8, ULVAC-PHI Inc., Kanagawa, Japan). The binding energy (BE) scale was corrected taking as reference the aliphatic component of C 1*s* at 284.8 eV. Other details about the analysis can be found elsewhere [15].

### 2.6. Colorimetric Quantification of Zn^2+^ Ionic Release from ZnO-Based Coatings

PEO coatings embedding CTAB-, PSS-, and PDDA-ZnOs were put in contact for 24 h with a buffered solution mimicking conditions adopted for the antiviral activity evaluation. MTEN buffer, consisting of MES (0.1 M), Tris (0.05 M), and EA (0.05 M), was selected as the contact medium and prepared at pH 7, adjusting the pH with NaOH 5 M. Its choice was dictated by its biocompatibility. Determination of Zn^2+^ ions was carried out following a previously reported method [16,17] using Zincon dye. The Zn^2+^ mother solution (0.1 M), used to get the standards for the assay by serial dilution, was prepared starting from ZnSO_4_·7H_2_O. MTEN buffer was used as the diluent for preparing Zn^2+^ standard solutions. Borate buffer at pH 9 was prepared with boric acid solution, adjusting the pH with NaOH 5 M. The pH of all buffers was checked by a bench meter HI5221 (Hanna Instruments, Padua, Italy).

For the colorimetric assay, 75 µL of a Zn^2+^ standard solution or sample (or MTEN buffer for blank) was mixed with 2.85 mL of borate buffer, and the reaction was initiated by adding 75 µL of Zincon solution (1.88 mM). UV/Vis spectra were recorded from 400 to 750 nm after 5 min of incubation using a Shimadzu UV-1601 double-beam spectrophotometer (Shimadzu Europa GmbH, Duisburg, DE). Absorbance at λ_max_ = 616 nm relevant to the Zn^2+^–Zincon complex was correlated to the concentration of zinc ions through a calibration curve using a blank and nine standards in the range 0–25 µM.

## 3. Results

### 3.1. Synthesis and Characterization of ZnONPs

ZnONPs were produced by means of sacrificial anode electrolysis, resulting in an electrochemical sol–gel approach. The main difference, compared to a conventional sol–gel method, resided in the electrochemical dosing of zinc ions in an alkaline environment, rather than using Zn(II) precursors/salts. A zinc working electrode acted as the source of Zn^2+^ in an acid carbonate aqueous solution used as electrolyte. The use of acid carbonate allows limiting zinc passivation, thus improving the yield of the zinc dissolution process.

**Figure 1:**
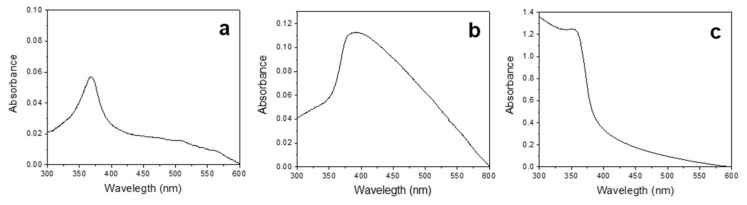
UV/Vis spectra of (**a**) CTAB-, (**b**) PDDA-, and (**c**) PSS-stabilized ZnONPs.

Stabilizing agents of different charge and nature (namely, CTAB, PDDA, and PSS) were also added to the electrolytic bath to generate mixed zinc carbonate and hydroxide species at low potentials (<1 V vs. RE). The presence of these species, which are typically employed in sol–gel synthesis to control the size and morphology of nanoparticles, were used here for the same purpose. The as-prepared electrosynthesized gels could then be dried and calcined (when needed) promoting the conversion of these nanostructured mixed species to ZnONPs (vide infra). The experimental yield of each electrochemical process was assessed by differential weighting of the electrodes before and after the synthesis. The theoretical mass was calculated according to Faraday’s law for a two-electron process [15]. Percentage yield was then obtained as a function of each stabilizer, which resulted equal to 79%, 70%, and 89% for PSS, PDDA, and CTAB, respectively. UV/Vis spectra (Figure 1) showed in all cases an absorbance peak around 370–380 nm (Figure 1a–c), in agreement with the known spectrophotometric behavior of zinc oxide [13,14,15].

**Figure 2:**
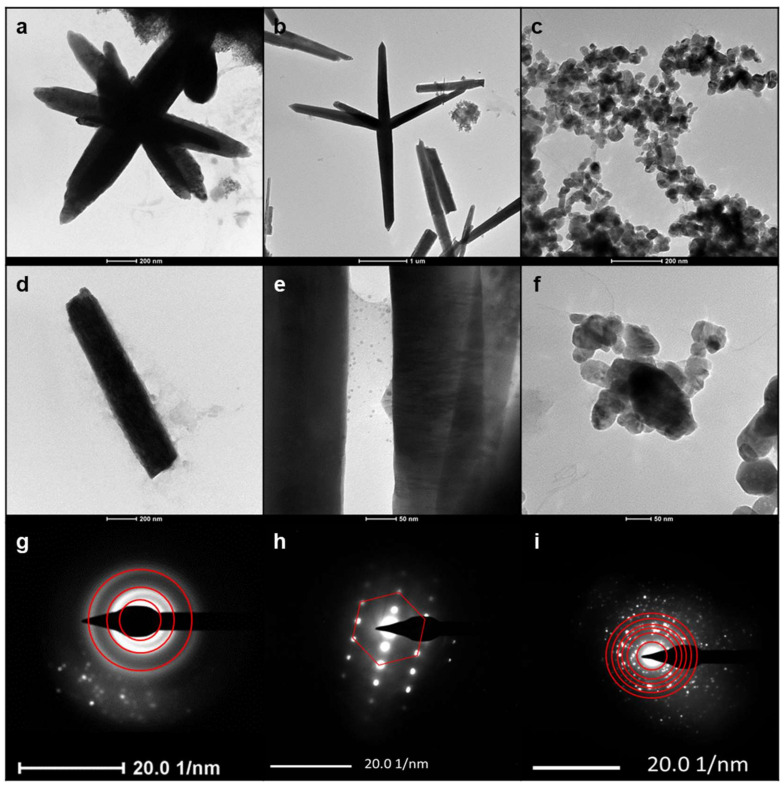
TEM micrographs of (**a**,**d**) CTAB-, (**b**,**e**) PDDA-, and (**c**,**f**) PSS-stabilized ZnONPs; SAED patterns of (**g**) CTAB-, (**h**) PDDA-, and (**i**) PSS-stabilized ZnONPs.

The NP surface charge was a function of the used stabilizer. Specifically, a positive ζ-potential value was measured for PDDA- and CTAB-capped ZnONPs (48 ± 3 mV and 38 ± 12 mV, respectively). PSS-ZnONPs showed, instead, a negative ζ-potential (–28 ± 4 mV). Morphological analyses on dried and annealed ZnONPs exhibited the influence of both stabilizers and thermal treatments. Figure 2 shows the TEM micrographs obtained on the ZnONPs prepared in the presence of different stabilizers. Elongated sub-micrometer rice-grain structures (with length around 1 μm) could be obtained preparing the colloids at 80 °C with CTAB and calcining them at 300 °C [14] (Figure 2a).

PDDA, after overnight drying at 120 °C, promoted the formation of wire-like microstructures, with a major length of 300–500 nm; growth lines moving along the wires suggested that these structures grew by lamellar superimposition [15] (Figure 2b). The use of PSS resulted in spheroidal NPs, with an average diameter around 50 nm, which aggregated in micron-sized clusters after calcination at 600 °C (Figure 2c). DLS measurements performed in buffered solution confirmed that agglomeration of calcined PSS-based ZnONPs occurred, since an average hydrodynamic diameter of about 2 µm was determined. On the other hand, CTAB- and PDDA-based NPs were less prone to aggregation as the average hydrodynamic diameter was comparable to the size determined by TEM analysis. In addition, sedimentation in about 10 min was determined by DLS characterization.

**Figure 3:**
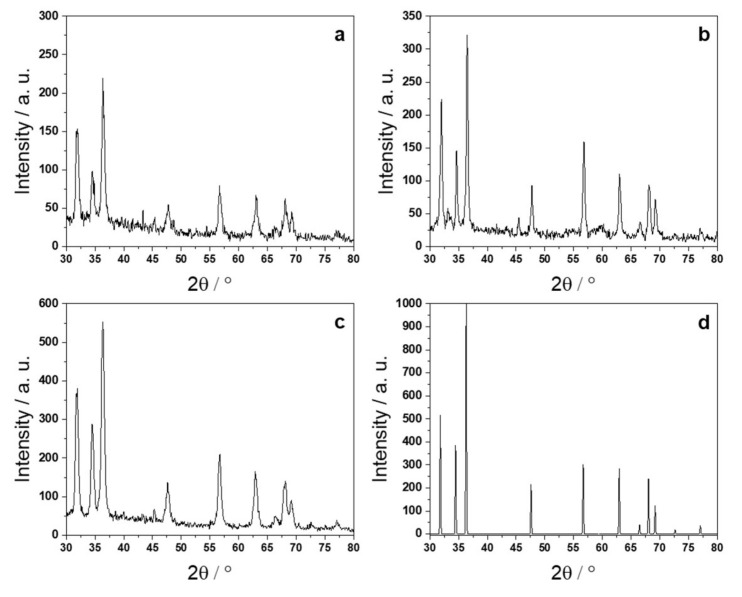
X-ray diffraction patterns of (**a**) CTAB-, (**b**) PDDA-, and (**c**) PSS-stabilized ZnONPs. In (**d**), we report, for comparison, the expected diffraction pattern for pure ZnO powders.

Returning to the morphological characterization, in all cases, ZnONPs showed the presence of interference fringes in high-magnification TEM micrographs (Figure 2d–f), thus evidencing their crystalline structure. This crystalline nature was confirmed by selected area electron diffraction (SAED) patterns obtained on the nanostructures (Figure 2g–i). CTAB-capped ZnONPs exhibited three diffused reflection rings, ascribable to (hkl) values of wurtzite, namely, 100, 002, and 102 [18]. The wire-like structure of PDDA-ZnO resulted in hexagonal SAED patterns, corresponding to the typical hexagonal structure of wurtzite [19]. Lastly, PSS-stabilized ZnONPs showed the presence of many reflections corresponding to the following (hkl) values of wurtzite, from inner to outer circle: 100, 002, 101, 102, 110, and 112 [18]. These findings were further corroborated by the XRD measurements reported in Figure 3. The diffraction peaks of the hexagonal form of ZnO dominated the patterns in all the cases. We have to remark that the PDDA-ZnO peak widths were sensibly narrower than in the PSS- and CTAB-ZnO spectra. This is evidence that the crystallites of PDDA-stabilized ZnO nanoparticles were of larger dimensions.

They also showed a preferential growth along the [001] direction. In panel (d) of Figure 3, we show for comparison the theoretical diffraction pattern of ZnO powder (database entry #197687) as obtained using the ICSD database (https://icsd.fiz-karlsruhe.de/index.xhtml). All the patterns measured for the different nanoparticles showed a remarkable agreement with the reference one. On the other hand, the small discrepancies in the scattering angle position, 0.1° or less, were simply due to imperfect centering of the sample with respect to the goniometer.

**Figure 4:**
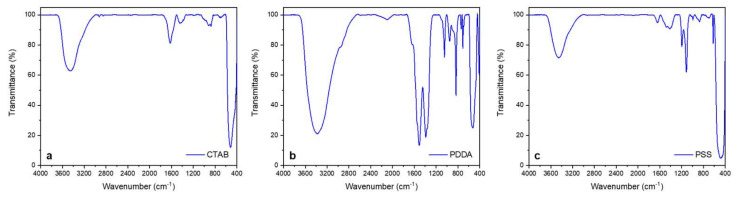
FTIR spectra of (**a**) CTAB-, (**b**) PDDA-, and (**c**) PSS-stabilized ZnONPs.

ATR-FTIR spectra of the three samples are reported in Figure 4. CTAB-stabilized ZnONPs (Figure 4a) exhibited an intense band below 500 cm^−1^, ascribable to Zn–O stretching [14]. Similar spectroscopic features were present in PSS-ZnONPs (Figure 4c) and are in agreement with the literature [13]. In these two cases, no IR bands below 3000 cm^−1^ typical of alkyl chains ascribable to the presence of the organic stabilizers were visible; this indicates that CTAB and PSS were completely degraded/removed during the calcination step. Regarding PDDA (Figure 4b), the IR spectrum of the dried sample presented some characteristic bands of PDDA, which was not degraded by the thermal treatment [15]. The presence of the two main bands relevant to the symmetric and asymmetric stretching vibrations of the C=O group at 1389–1500 cm^−1^ and of the other characteristic signals at 1055 cm^−1^ and 842 cm^−1^, combined with the presence of the 3400 cm^−1^ band relative to O–H stretching, indicates that the milder thermal treatment resulted in an uncomplete decomposition of Zn^2+^ carbonates and hydroxides.

### 3.2. Antiviral Activity of ZnONPs against SARS-CoV-2

Nasopharyngeal swabs were collected from patients positive for SARS-CoV-2 infection and tested with the CLEIA SARS-CoV-2 antigenic test. The initial sample concentration of SARS-CoV-2 nucleocapsid (N) protein antigen was quantitatively measured, and sample serial dilutions were prepared in DPBS. Each dilution was exposed to ZnONPs (CTAB-, PDDA-, PSS-capped ones). After 24 h of contact, SARS-CoV-2 N protein antigen was quantified again, and the percentage concentration decrease was calculated. The results are summarized in Table 1.

Table 1 shows that higher decreasing percentages were obtained, as expected, when 10 g/L of ZnONPs are used. PDDA-ZnONPs were able to reduce antigenic dosage to a higher extent, with respect to PSS- and CTAB-ZnONPs. This is quite surprising considering that PSS-ZnONPs have a lower mean size, i.e., a higher surface/volume ratio, which is in general correlated to a higher antimicrobial activity [20]. Additionally, it is known that the SARS-CoV-2 N protein contains more positively charged residues and less negatively charged residues with respect to those of other CoVs (SARS-CoV, in particular) which may lead to an increased affinity to bind to negatively charged regions of other molecules through nonspecific polar interactions [21,22,23]. Hence, the presence of a high number of positive residues should lay behind a higher antiviral activity exerted by negatively charged PSS-ZnONPs. However, we can infer that the wire-like structure of PDDA allows for a more controlled and long-lasting ionic release in solution, while particles with an aspect ratio closer to one (as per PSS- and CTAB-capped ones) show a faster and less controlled release in unsaturated aqueous medium [24]. A certain mechanical action due to the presence of elongated and sharp structures can also be inferred for PDDA-ZnONPs [25,26]. This could explain the higher abatement of antigen concentration in physiologic solution, over a 24 h timespan, for PDDA-ZnONPs. We also found evidence of a certain dilution dependance of the inhibitory effect, at comparable antigen concentrations (Table S1). Human mucus is mainly composed of water (around 95%); furthermore, mainly glycoproteins are present (between 2% and 5%), in addition to proteins, lipids, nucleic acids, gluconic acid derivatives, etc. to a lower extent (below 0.5%) [27]. A higher sample dilution (i.e., above 1:10) always resulted in a higher antigen reduction after contact with ZnONPs for 24 h. It is known in the literature that double-charged metal ions, such as Zn^2+^, have a high affinity for mucosal glycoproteins [28,29]. Specifically, protein cysteines can act as coordination sites for zinc ions, and zinc gluconates may be formed. A higher sample dilution, resulting in a lower presence of mucus components in solution, leads to a higher amount of Zn^2+^ ions in contact medium, which are not sequestered by mucus biomolecules, free to exert their antiviral activity on the SARS-CoV-2 N protein. The affinity of the N protein for Zn^2+^ metal ions has already been theoretically and experimentally determined [30,31]; hence, our results can be considered coherent with the literature findings and can hint at the potential antiviral role of Zn^2+^-releasing materials in effectively fighting COVID-19 transmission. Additionally, a recent report hypothesized virus inactivation by Zn^2+^ release and ROS formation. Specifically, four mechanisms were described as the most probable: (i) ZnO nanostructures for the possible anchoring of SARS-CoV-2 virions, thus inhibiting interaction with host cell receptors; (ii) internalization of ZnO nanostructures for the inhibition of early stages of the viral replication cycle; (iii) ion release as a surface attack mechanism to disrupt the plasmid and RNA virus integrity; (iv) photocatalytic generation of reactive oxygen species for the possible degradation of the lipid, protein, and nucleic structure of SARS-CoV-2 [32].

It is worth noting that, for PDDA-ZnO, the stabilizer does not play any role in the antiviral activity; this polymer, in fact, despite its known antibacterial activity (as a quaternary ammonium compound [33]), does not inhibit or limit the viral lifecycle. On the contrary, it has been reported to inhibit the antiviral properties of other active materials, such as graphene oxide against porcine epidemic diarrhea RNA virus [34].

**Figure 5:**
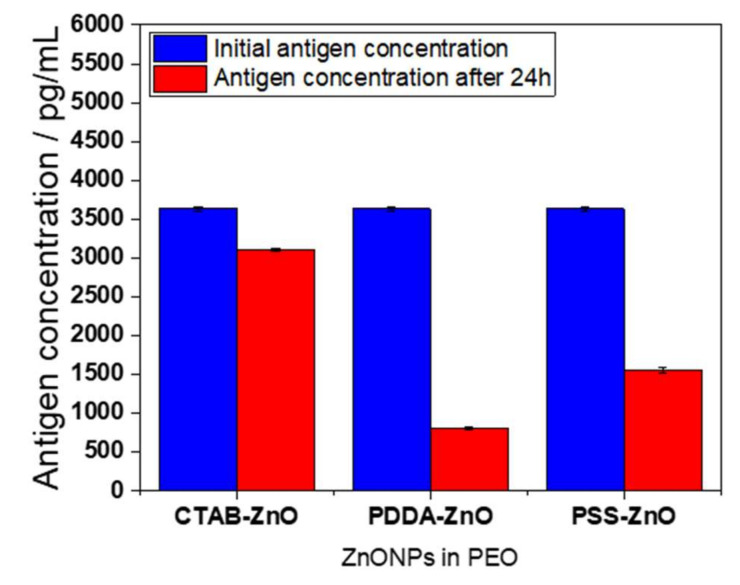
Effectiveness of composite coatings based on PEO, embedding CTAB-, PDDA-, and PSS-capped ZnONPs. Antigen quantification was performed after 24 h of contact. Data were averaged across three replicates, with corresponding standard deviations. Control experiments on bare PEO did not show any change in antigen quantification.

On the basis of these results, we decided to exploit the antiviral activity of ZnONPs to produce ZnO-based coatings for hard touching surfaces. We embedded ZnONPs in PEO; this polymer was chosen because it is biodegradable, nontoxic, and approved by the Food and Drug Administration for use in food and pharmaceutical products [35]. We tested the highest viral loading available on a modified silicon slide (contact area ~100 mm^2^), for 24 h contact. A starting antigen dosage of about 3600 pg/mL was lowered by the active coating, as displayed in Figure 5. PDDA-ZnO-based composites were, as expected from measurements on loose powders, the most effective ones. Considering that ZnONPs are embedded into the polymeric matrix, which limits their active surface to exert ionic release and consequent antiviral activity, these results are a promising starting point for further investigations. We believe that this coating could offer a good self-cleaning strategy to ensure surface disinfection. The contamination of commonly touched surfaces can be regarded as responsible for many contagion episodes, both in nosocomial environments and in public places [36]. The usage of bioactive nanoparticles as polymer-confined nano-reservoirs, providing a source of ionic release, without being released as entire nanostructures in the surrounding environment, is a key issue in our laboratories. Blocking antiviral ZnONPs in a toxicologically safe embedding matrix could be, in our opinion, the best way to guarantee an appropriate antiviral action on touching surfaces.

In order to better understand the reasons behind the different efficacy of the three composite coatings, both the elemental Zn surface availability and the amount of the Zn^2+^ ionic release were evaluated by X-ray photoelectron spectroscopy (XPS) and visible spectroscopy, respectively. Table 2 summarizes these data. Moreover, the overall surface composition of the coatings, as per the complete XPS analysis, is reported in the Table S2.

A good correlation was found between the ionic release exerted by the coatings and the antiviral activity; the highest Zn^2+^ ionic release led to the highest inhibition efficiency. Contrarywise, the Zn elemental surface availability, quantified by XPS, showed a fairly opposite trend.

The different zinc elemental percentages found on the surface of the different coatings can be explained on the basis of the different location of the particles in the outer layers of the coating surface. We should consider that XPS has a sampling depth of a few nanometers, whereas the average size of CTAB- and PDDA-capped ZnO is typically in the sub-micrometer range. The coatings were obtained using the same particle/polymer mixing ratio; hence, the differences in the third column of Table 2 should be interpreted exclusively in terms of different surface segregation.

The different extent of the ionic release exerted by the different coatings is also not surprising, due to the totally different chemical nature and size of the particles. PDDA-ZnO NPs did not undergo any calcination step, thus preserving the chemical nature of the stabilizer unaltered [15]. CTAB- and PSS-capped ZnO particles underwent thermal treatments (for obtaining stoichiometric ZnO), and this affected their shell, which at least partially lost its molecular structure (see the absence of Br in the second row of Table S2).

The hydrophilic nature of the PEO dispersing matrix, which is known to undergo water swelling [37], allowed the different materials to provide their ionic release independently of the particle location in the outer surface layers.

Under these circumstances, it is evident that contact effects, involving the surface of the abovementioned coatings, did not affect significantly their antiviral efficacy. This is also reasonable considering that the well-known photocatalytic and ROS-generating properties of ZnONPs were not exploited in this study, which was carried out without the assistance of UV irradiation.

The results of Table 2 clearly demonstrate that it is possible to correlate the effectiveness of the composite coatings with the entity of ionic release, whereas ZnONPs/virus contact effects have minor relevance under the present experimental conditions.

It is worth pointing out that the maximum concentration of Zn^2+^ reached in this study is lower than the NOAEL (*no observed adverse effect level*) safety limit reported by the EU [38] and USA [39] authorities for skin contact.

## 4. Conclusions

Antiviral ZnONPs were produced via galvanostatic routes in the presence of different stabilizers; the nanomaterials were characterized from a morphological and spectroscopic point of view. Antiviral activity of ZnONPs was tested by antigen quantification on real SARS-CoV-2 samples from nasopharyngeal swabs. ZnONPs were demonstrated to strongly lower SARS-CoV-2 antigen dosage, with percentage values up to 90%. The preparation of ZnONPs–PEO composites was also studied, and preliminary tests proved the high efficacy of this material as an effective self-cleaning coating for hard surfaces exposed to SARS-CoV-2 infection. Due to their nontoxic and straightforward handy nature, the aforementioned composites could be easily brushed on commonly touched surfaces and left to dry. Work is in progress on the combination of ion-releasing properties and UV irradiation, in order to further increase the efficacy of our ZnONPs in reducing the persistency of the target virus. Moreover, we are planning to test our composite materials on SARS-CoV-2 using common titer tests on infected human cell lines; different polymer matrices could also be used, ranging from common materials for food packaging to biomedical ones.

## Figures and Tables

**Table 1:** Antigen quantification before and after exposure to 10 g/L or 1 g/L ZnONPs.

Sample Type	Dilution Factor	Initial (pg/mL)	Final (pg/mL)	Decreasing %	Mean (pg/mL)	Decr. Mean %
ZnO/PSS 10 g/L	1:10	3634.9 ± 0.5	2363.3 ± 0.5	35.0	2436 ± 72	33 ± 2
			2507.8 ± 0.5	31.0		
ZnO/PSS 1 g/L			2831.5 ± 0.5	22.1	2695 ± 136	26 ± 4
			2559.1 ± 0.5	29.6		
ZnO/PDDA 10 g/L	1:10	3634.9 ± 0.5	385.5 ± 0.5	89.4	375 ± 11	89.7 ± 0.3
			363.9 ± 0.5	90.0		
ZnO/PDDA 1 g/L			1187.3 ± 0.5	67.3	1257 ± 70	65.4 ± 1.9
			1327.6 ± 0.5	63.5		
ZnO/CTAB 10 g/L	1:10	3634.9 ± 0.5	2649.7 ± 0.5	27.1	2651.5 ± 1.8	27.0 ± 0.1
			2653.3 ± 0.5	27.0		
ZnO/CTAB 1 g/L			2713.4 ± 0.5	25.4	2895 ± 319	20 ± 5
			3075.7 ± 0.5	15.4		

**Table 2:** Correlation among the Zn^2+^ ionic release in solution (after 24 h of contact, second column), the zinc surface atomic percentage (Zn%, as determined by XPS analysis, third column), and the inhibition efficiency (estimated through the N protein antigen quantification, last column). Data are reported as mean values ± 1 standard deviation over three independent experiments.

Sample	[Zn^2+^]/mg/L (in 24 h)	Zn%	Inhibition %
CTAB-ZnO in PEO	4.2 ± 0.6	1.7 ± 0.2	14 ± 3
PDDA-ZnO in PEO	45 ± 4	0.5 ± 0.2	78 ± 2
PSS-ZnO in PEO	13 ± 1	6.6 ± 0.7	57 ± 3

## Data Availability

Not applicable.

## Supplementary Materials

The following supporting information can be downloaded at https://www.mdpi.com/article/10.3390/ijms23063040/s1.
