# Supplementary material for: On the Efficacy of ZnO Nanostructures against SARS-CoV-2
Source: Int J Mol Sci. 2022 Mar 11;23(6):3040. doi: 10.3390/ijms23063040 (PMC8950216; doi:10.3390/ijms23063040)
Supplement: Supplementary file 1 [file ijms-23-03040-s001.zip › ijms-1622845-supplementary.pdf]

Supporting Information

On the efficacy of ZnO nanostructures against SARS-CoV-2

Maria Chiara Sportelli[1‡], Margherita Izzi[1‡], Daniela Loconsole[2], Anna Sallustio[3], Rosaria Anna Picca[1], Roberto Felici[3], Maria Chironna[2], Nicola Cioffi[1*]*

1. Chemistry Department, University of Bari Aldo Moro, V. Orabona, 4, 70126, Bari, Italy.

2. Department of Biomedical Sciences and Human Oncology-Hygiene Section, University of Bari Aldo Moro, Piazza Giulio Cesare, 11 70124, Bari, Italy.

3. Azienda Ospedaliero-Universitaria Consorziale Policlinico di Bari, P.zza G. Cesare 11, 70124, Bari, Italy

4. CNR-SPIN, Area della Ricerca di Tor Vergata, Via del Fosso del Cavaliere, 100, 00133 Rome, Italy

Table S1. Antigen quantification before and after exposure to 10 g/L ZnONPs. Effect of sample dilution in DPBS is evident in antigen decreasing percentages, which are higher when the real sample is more diluted (at comparable initial antigen concentrations). [a]SD = standard deviation calculated on three replicates; [b]u. = unmeasurable, i.e. below the limit of quantification (LoQ = 0.05 pg/mL).

**Sample type**	**Dilution factor**	**Initial (pg/mL)**	**Final (pg/mL)**	**Decreasing %**	**Mean ± SD[a] (pg/mL)**	**Decr. Mean % ± SD[a]**
ZnO/PDDA 10 g/L	1:10	4062.7 ± 0.5	1195.3 ± 0.5	70.6	1297 ± 88	68 ± 2
			1343.9 ± 0.5	66.9		
			1350.5 ± 0.5	66.8		
	1:100	346.6 ± 0.5	53.7 ± 0.5	84.5	56 ± 4	83.9 ± 1.0
			60.1 ± 0.5	82.7		
			53.9 ± 0.5	84.4		
	1:1000	29.1 ± 0.5	2.1 ± 0.5	92.8	2.6 ± 0.4	91.2 ± 1.4
			2.8 ± 0.5	90.3		
			2.7 ± 0.5	90.6		
ZnO/PSS 10 g/L	1:10	4062.7 ± 0.5	646.4 ± 0.5	84.1	625 ± 52	84.6 ± 1.3
			662.7 ± 0.5	83.7		
			564.9 ± 0.5	86.1		
	1:100	346.6 ± 0.5	90.2 ± 0.5	74.0	73 ± 16	80 ± 5
			70.8 ± 0.5	79.6		
			58.0 ± 0.5	83.3		
	1:1000	29.1 ± 0.5	4.0 ± 0.5	86.3	10 ± 9	65 ± 30
			20.4 ± 0.5	30.0		
			6.4 ± 0.5	78.1		
ZnO/CTAB 10 g/L	1:1000	4712.9 ± 0.5	21.9 ± 0.5	99.5	22 ± 2	99.5 ± 0.1
			19.5 ± 0.5	99.6		
			24.0 ± 0.5	99.5		
	1:10000	359.9 ± 0.5	1.2 ± 0.5	99.7	0.7 ± 0.4	99.8 ± 0.1
			0.7 ± 0.5	99.8		
			0.4 ± 0.5	99.9		
	1:100000	29.6 ± 0.5	u.[b]	100	0	100
			u.[b]	100		
			u.[b]	100		

Table S2. XPS surface chemical composition (expressed as atomic percentages At%) of PEO coatings embedding CTAB-, PDDA-, and PSS-ZnO. Values represent means ± 1 standard deviation of three independent experiments.

**Sample**	**C%**	**O%**	**Zn%**	**N%**	**Na%**
**CTAB-ZnO/PEO**	59.8 ± 1.1	38.5 ± 1.2	1.7 ± 0.2	-	-
**PDDA-ZnO/PEO**	65.0 ± 0.7	33.3 ± 0.5	0.5 ± 0.2	0.7 ± 0.5	≤ 0.5
**PSS-ZnO/PEO**	52.3 ± 1.6	40.2 ± 0.7	6.6 ± 0.7	-	0.9 ± 0.5
